# Association between miRNA-499 gene polymorphism and autoimmune diseases: A meta-analysis

**DOI:** 10.1371/journal.pone.0266265

**Published:** 2022-03-31

**Authors:** Xiangjian Kong, Shuling Diao, Huipu Xu, Junming Sun, Baoxin Ma

**Affiliations:** 1 Department of Cardiology, The Affiliated Hospital of Binzhou Medical University, Binzhou, Shandong, China; 2 Second Department of Cardiology, Central Hospital Affiliated to Shandong First Medical University, Jinan, Shandong, China; Unicamillus, Saint Camillus International University of Health Sciences, ITALY

## Abstract

**Introduction:**

The association between miRNA-499 rs3746444 and a variety of autoimmune diseases has been reported. However, these results were contradictory and just focused on one or two autoimmune diseases. The present study aims to examine the possible association between rs3746444 polymorphism and the risk of autoimmune diseases.

**Methods:**

The studies that evaluated the association between miRNA-499 gene polymorphism and autoimmune diseases were retrieved. Five different genetic models were used to evaluate the association. The random-effects model was used to pool the effect sizes. Odds ratios (ORs) and 95% confidence intervals (CIs) were calculated to estimate the associations. Stratification analyses were performed by ethnicity and type of autoimmune diseases. False-positive report probability (FPRP) was performed for determining noteworthy associations.

**Results:**

Seventeen articles (twenty studies) involving 4,376 cases and 4,991 controls were identified and included in our meta-analysis. The pooled ORs of all eligible case-control studies indicated a significant association between miRNA-499 gene polymorphism and autoimmune diseases: (T vs. C: OR = 0.877; 95% CI: 0.774, 0.993; P = 0.039). Stratified analysis indicated a significant association across both Caucasian (TT vs. TC+CC: OR = 0.779; 95% CI: 0.622, 0.976; P = 0.030) and Asian (T vs. C: OR = 0.895; 95% CI: 0.808, 0.992; P = 0.035) populations. There was also a significant association in Behcet’s disease, rheumatoid arthritis, systemic lupus erythematosus, and ulcerative colitis populations.

**Conclusions:**

Our meta-analysis suggested that the miRNA-499 rs3746444 polymorphism was associated with an elevated risk of autoimmune diseases in the overall analysis as well as Caucasian and Asian populations.

## 1. Introduction

MicroRNAs (miRNAs) are small non-coding RNAs of 19 to 25 nucleotides, and their function is to regulate the expression of their target gene [1, 2]. It has been proposed that miRNAs act by binding to the 3’ -UTR of target mRNA, regulating the expression of protein-coding genes [3, 4].

MiRNAs are present in many biological fluids and can regulate a broad range of physiologic and pathologic processes [2]. Variants of miRNAs are diagnostic biomarkers of several diseases [5–7]. Many studies have announced that genetic variations play a vital part in the occurrence and progression of autoimmune diseases [8–10].

An association between miRNA-499 rs3746444 and a variety of autoimmune diseases including rheumatoid arthritis [11], systemic lupus erythematosus [12], Graves’ disease [12], and Ankylosing Spondylitis [13] have been reported. However, The experimental data are rather controversial, and there is no general agreement about the association between miRNA-499 rs3746444 and autoimmune diseases. Several meta-analyses assessing the association between miRNA-499 rs3746444 and autoimmune disease risk were published before 2021 [14–18]. But they always pay attention to one or two autoimmune diseases. We included as many autoimmune diseases as possible and included related articles newly published in recent years. The number of studies was significantly greater than that in other meta-analyses published before. This could increase the statistical power in the overall analysis.

The present study aimed to examine the possible association between rs3746444 polymorphism and the risk of autoimmune diseases.

## 2. Material and methods

### 2.1 Inclusion and exclusion criteria

Case-control studies included in this study met these criteria: i evaluated the association between miRNA-499 gene polymorphism and autoimmune diseases; ii available and sufficient data including the distribution of genotype frequency in case and control groups. The study exclusion criteria were: i review papers, editorials, comments; ii studies without controls; iii not provided enough information to calculate the odds ratios (ORs) and 95% confidence interval; iv Hardy-Weinberg Equilibrium (HWE) <0.05 in the control group.

### 2.2 Bibliographic search

PubMed, Embase, Scopus, Web of Science, Wanfang, and Chinese National Knowledge Infrastructure databases were searched with the full electronic search strategy as follows: (“microRNA-499” OR “miRNA-499” OR “rs3746444” OR “Pre-miR-499”) AND (“gene” OR “Genetic Polymorphism” OR “Polymorphism” OR “genetic” OR “allele” OR “variation” OR “variant” OR “mutation”) AND (“autoimmune diseases” OR “autoimmune disease” OR “arthritis, rheumatoid” OR “rheumatoid arthritis” OR “lupus erythematosus, systemic” OR “systemic lupus erythematosus” OR “psoriasis” OR “Sjogren’s syndrome” OR “Behcet’s disease” OR “Vogt–Koyanagi–Harada disease” OR “systemic sclerosis” OR “multiple sclerosis” OR “primary antiphospholipid syndrome” OR “Addison’s disease” OR “Diabetes Mellitus, Type 1” OR “graves disease” OR “juvenile idiopathic arthritis” OR “ankylosing spondylitis” OR “polymyositis” OR “dermatomyositis” OR “myasthenia gravis”). The last search was performed on December 15, 2021. We also searched for additional pertinent studies through the references of all identified publications. There was no language restriction in the literature search.

### 2.3 Extraction of data

Two authors independently extracted the following data from the selected studies: first author; year of publication; country; ethnicity; sample size; genotype frequencies; type of autoimmune diseases; Genotyping methods; Hardy–Weinberg equilibrium(HWE) for controls. Study quality was assessed according to the Newcastle-Ottawa quality-assessment scale. Quality scores ranged from 0 to 9. The work of extraction of data was operated by 2 independent researchers (Kong and Ma). Any disagreement was discussed and resolved with a third author (Xu).

### 2.4 Statistical analysis

The main meta-analysis compared the presence of miRNA-499 gene polymorphism among patients with autoimmune diseases as cases versus healthy subjects as controls. We assessed HWE via Chi-square test in the control populations. The association between miRNA-499 gene polymorphism and autoimmune diseases was estimated by odds ratios (ORs) and 95% confidence intervals (CIs). The strength of the association was determined based on five different genetic models: allelic model (T vs. C), heterozygote model (TC vs. CC), homozygote model (TT vs. CC), dominant model (TT vs. TC+CC), recessive model (TT+TC vs. CC). Because the random-effects model (DerSimonian and Laird method) can incorporate a heterogeneity parameter and enable the modeling of differences between studies, we used it to pool the effect sizes in our analysis [19, 20]. A P-value<0.05 was considered statistically significant. Stratification analyses were performed by ethnicity and type of autoimmune diseases. Sensitivity analysis was applied to assess the stability of the results by omitting each study in each turn. Furthermore, we used Begg’s funnel plot [21] and egger’s regression test [22] to assess the publication bias within the studies. The false-positive report probability (FPRP) values at different prior probability levels for all significant findings were assessed [23]. An FPRP value <0.2 represented a noteworthy association. Meta-analysis was carried out using the STATA version 12.0 software (Stata Corporation, College Station, TX, USA).

## 3. Results

### 3.1. Selection of eligible studies

**Fig 1:**
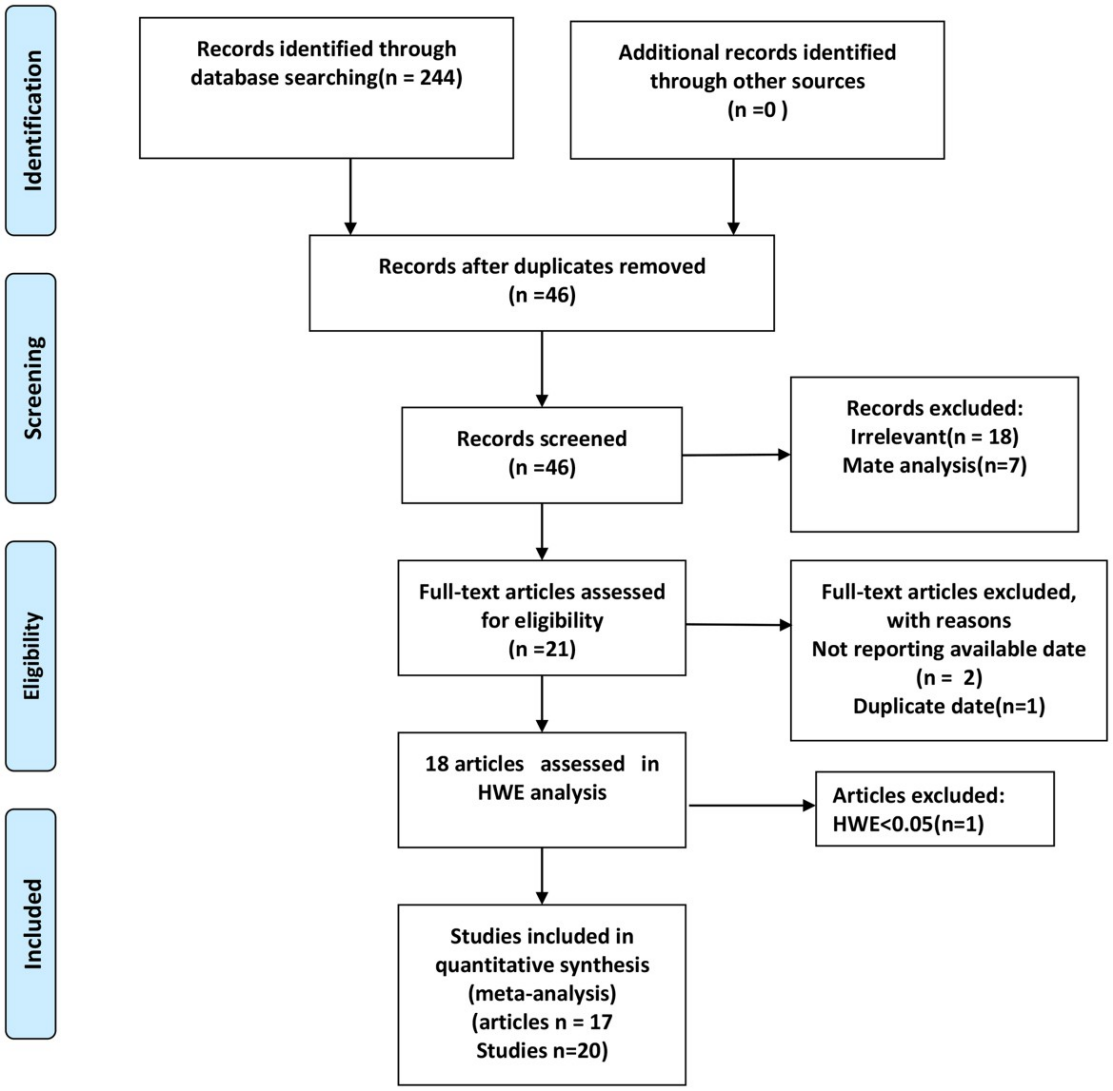
Flow diagram of study selection.

Our search initially yielded a total of 244 potential articles (Fig 1). After the removal of duplicates, 46 articles were selected for further analysis. After reviewing the titles and abstracts, 25 of these 46 articles were excluded because of the lack of relevant results and the type of articles. 21 full-text articles were assessed for eligibility. 3 articles were excluded due to the non-reporting of available data and duplicate data. 18 articles were assessed in HWE analysis. The genotype distribution in the controls was compatible with the HWE in 17 articles (20 studies) [11–13, 24–37], which were ultimately included in our analysis. The basic characteristics of included studies are represented in Table 1.

**Table 1:** The main characteristic of studies included in the meta-analysis.

NOS	8	8	8	8	8	5	7	7	5	5	5	7	8	7	8	8	8	8	7	8
HWE of control	0.495	0.495	0.88	0.839	0.742	0.696	0.837	0.15	0.458	0.458	0.458	0.124	0.807	0.165	0.07	0.841	0.719	0.624	0.214	0.055
C(case/ control)	23/101	23/101	55/43	110/86	81/61	44/56	195/168	165/328	672/62	63/62	12/62	299/233	109/141	93/167	24/31	115/86	50/130	56/63	62/57	74/151
T(case/ control)	155/373	77/373	49/69	98/138	119/139	50/44	225/256	229/554	743/910	761/910	150/910	1751/1583	663/1011	107/123	180/179	319/404	362/802	360/417	364/361	266/655
CC(case/ control)	0/9	0/9	16/8	32/16	14/10	7/15	50/34	65/54	0/1	3/1	0/1	17/20	5/8	14/44	1/0	11/8	3/10	7/5	6/6	6/20
TC(case/ control)	23/83	23/83	23/27	46/54	53/41	30/26	95/100	35/220	71/60	57/60	12/60	265/193	99/125	65/79	22/31	93/70	44/110	42/53	50/45	62/111
TT(case/ control)	66/145	27/145	13/21	26/42	33/49	10/9	65/78	97/167	336/425	352/425	69/425	743/695	282/443	21/22	79/74	113/167	159/346	159/182	157/158	102/272
Sample size (case/control)	89/237	50/23	52/56	104/112	100/100	47/50	210/212	197/441	407/486	412/486	81/486	1025/908	386/576	100/145	102/105	217/245	206/466	208/240	213/209	170/403
Genotyping methods	(T-ARMS-PCR)	(T-ARMS-PCR)	RT- PCR	TaqMan	PCR-RFLP	TaqMan	PCR-RFLP	RT- PCR	TaqMan	TaqMan	TaqMan	PCR	TaqMan	PCR-RFLP	PCR	PCR-RFLP	MALDI-TOF	PCR-RFLP	PCR-RFLP	PCR-RFLP
Disease	RA	SLE	RA	RA	RA	BD	UC	UC	SLE	RA	GD	AITD	RA	BD	AS	RA	RA	RA	SLE	UC
Ethnicity	Caucasian	Caucasian	Caucasian	Caucasian Caucasian Caucasian	Caucasian Caucasian Caucasian	Caucasian Caucasian Caucasian	Caucasian Caucasian Caucasian	Asian	Caucasian	Caucasian	Caucasian	Asian	Asian	Caucasian	Asian	Caucasian	Asian	Asian	Asian	Asian
country	Iran	Iran	Egyptian	Egypt	Egypt	Egypt	Iran	India	Mexico	Mexico	Mexico	China	China	Turkey	China	Egypt	China	China	China	Japan
Year	2020	2020	2018	2018	2018	2017	2017	2017	2017	2017	2017	2017	2016	2015	2015	2013	2013	2011	2011	2011
Author	Ahmadi 1	Ahmadi 2	Ayeldeen	Shaker	Fattah	Eissa	Ghobadi	Ranjha	Alemán-Ávila 1	Alemán-Ávila 2	Alemán-Ávila 3	Cai	Yang	Oner	Xu	EI-Shal	Zhang	Yang	Zhang	Okubo

Rheumatoid arthritis (RA), Behcet’s disease (BD), Ulcerative colitis (UC), Systemic lupus erythematosus (SLE), Graves’ disease (GD), Autoimmune thyroid diseases (AITDs), Ankylosing spondylitis (AS).

Among these studies, eight studies were carried out in the Asian population [13, 25, 29, 31–33, 36, 37]. Nine articles(twelve studies) were conducted on Caucasians [11, 12, 24, 26–28, 30, 34, 35]. There are two articles focused on different diseases Simultaneously in one writing. One study focused on two different types of diseases including rheumatoid arthritis (RA) and systemic lupus erythematosus (SLE) [24] and another study by Alemán-Ávila et al. focused on three different types of diseases including rheumatoid arthritis (RA), Graves’ disease (GD) and systemic lupus erythematosus (SLE) [12]. So finally nine studies were related to rheumatoid arthritis (RA) [11, 12, 24, 27, 31, 33–36], three to ulcerative colitis (UC) [28, 29, 37], three to systemic lupus erythematosus (SLE) [12, 24, 32] and two studies were related to Behcet’s disease (BD) [26, 30]. There is one article each related to autoimmune thyroid diseases [25], Graves’ disease (GD), and ankylosing spondylitis (AS) [13].

### 3.2 Meta-analysis comparing the association between any systemic autoimmune disease and miRNA-499 gene polymorphism

**Fig 2:**
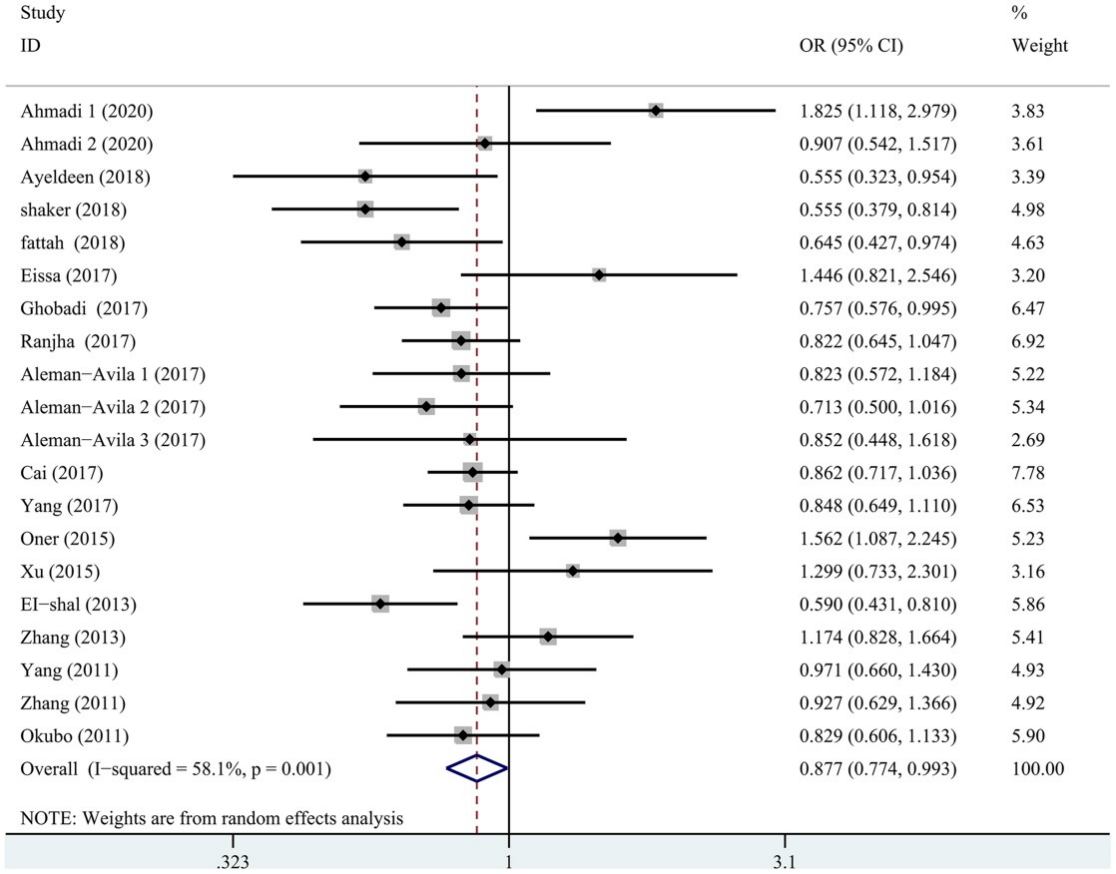
Meta-analysis of the association of the miRNA-499 rs3746444 polymorphisms with the autoimmune diseases (T vs. C).

**Fig 3:**
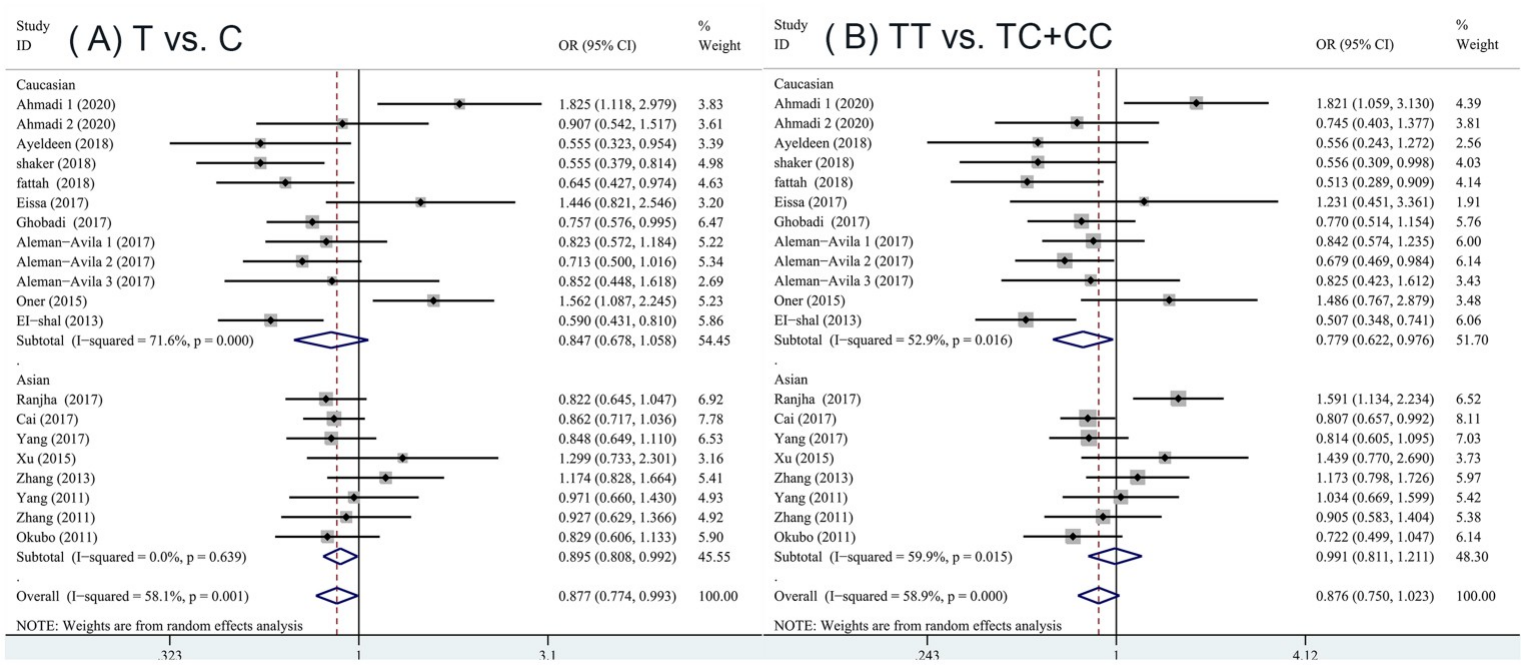
Stratified analysis of the association of the miRNA-499 rs3746444 polymorphisms with the autoimmune diseases by ethnicity. (A) T vs. C, (B) TT vs. TC+CC.

Table 2 summarized the results of the meta-analysis for a possible association between any systemic autoimmune disease and miRNA-499 gene polymorphism. The pooled ORs of all eligible case-control studies indicated a significant association: (T vs. C: OR = 0.877; 95% CI: 0.774, 0.993; P = 0.039)(Fig 2). An ethnicity analysis further showed that among Asians, a significant association was observed under some genetic model (T vs. C: OR = 0.895; 95% CI: 0.808, 0.992; P = 0.035). In the Caucasian population, there was also a significant association between systemic autoimmune diseases and miRNA-499 gene polymorphism (TT vs. TC+CC: OR = 0.779; 95% CI: 0.622, 0.976; P = 0.030)(Fig 3).

**Table 2:** Meta-analysis of miRNA-499 rs3746444 and autoimmune diseases.

rs3746444	No	Genetic model	Association test		Heterogeneity		Prior Probability	
			OR(95% CI)	*p*	I^2^(%)	*p*_H_	0.25	0.1
Overall	20	T vs. C	**0.877(0.774–0.993)**	**0.039**	71.2	0.030	0.103	0.257
	20	TT vs. CC	0.804(0.570–1.135)	0.216	50.3	0.006	0.429	0.693
	20	TC vs. CC	0.927(0.563–1.527)	0.765	77.1	<0.001	0.718	0.884
	20	TT+TC vs. CC	0.860(0.575–1.285)	0.460	68.1	<0.001	0.608	0.823
	20	TT vs. TC+CC	0.876(0.750–1.023)	0.095	58.9	<0.001	0.211	0.459
Ethnicity								
Caucasian	12	T vs. C	0.847(0.678–1.058)	0.144	71.6	0.001	0.305	0.568
	12	TT vs. CC	0.775(0.435–1.380)	0.387	61.6	0.003	0.625	0.833
	12	TC vs. CC	1.000(0.605–1.652)	0.999	56.3	0.009	N/A	N/A
	12	TT+TC vs. CC	0.882(0.514–1.515)	0.649	65	0.001	0.697	0.874
	12	TT vs. TC+CC	**0.779(0.622–0.976)**	**0.030**	52.9	0.016	0.090	0.228
Asian	8	T vs. C	**0.895(0.808–0.992)**	**0.035**	0.0	0.639	0.094	0.237
	8	TT vs. CC	0.841(0.574–1.231)	0.372	25.2	0.228	0.559	0.792
	8	TC vs. CC	0.797(0.304–2.090)	0.645	86.4	<0.001	0.751	0.900
	8	TT+TC vs. CC	0.838(0.438–1.602)	0.593	72.9	0.001	0.702	0.876
	8	TT vs. TC+CC	0.991(0.811–1.211)	0.928	59.9	0.015	0.736	0.893

**Fig 4:**
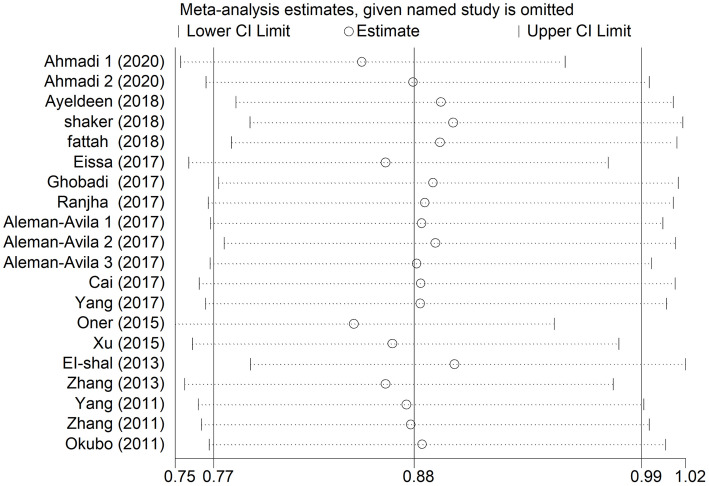
Sensitivity analysis of each study included in this meta-analysis was performed by omitting each data set from the analysis (T vs. C).

**Fig 5:**
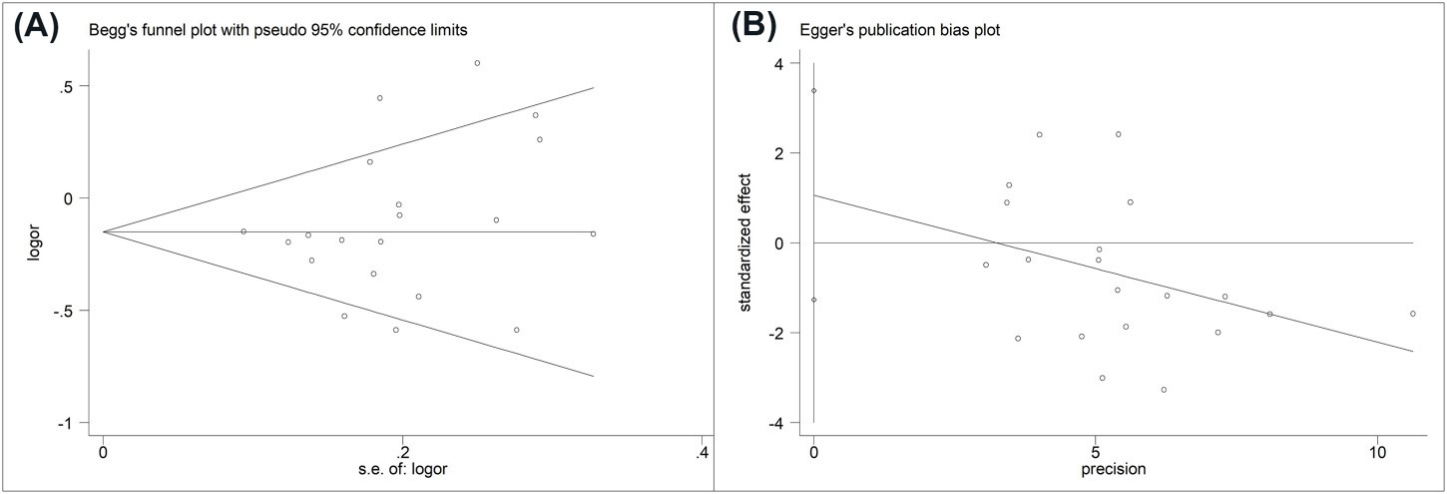
Funnel plot to assess the risk of publication bias in the meta-analysis. (A) Begg’s funnel plot, (B) Egger’s publication bias plot.

A sensitivity analysis was applied to assess the influence of each study on the pooled ORs. When the association between any systemic autoimmune disease and miRNA-499 gene polymorphism was analyzed in model T vs. C, the exclusion of each study showed a significant association(Fig 4). The results were stable. Furthermore, we used Begg’s funnel plot and egger’s regression test to assess the publication bias within the studies included in the meta-analysis. No significant publication bias was found in all genetic models (T vs. C: Begg: P = 0.456, egger: P = 0.352)(Table 3 and Fig 5).

**Table 3:** The publication bias in the meta-analysis.

Genetic model	Begg’s test		Egger’s test	
	Z	*p*	t	*p*
T vs. C Total	0.75	0.456	0.96	0.352
TT vs. CC Total	0.94	0.347	1.39	0.181
TC vs. CC Total	0.19	0.846	1.48	0.157
TC vs. CC Total	0.36	0.721	1.68	0.11
TT vs. TC+CC Total	0.75	0.456	0.41	0.686

### 3.3 Meta-analysis comparing specific systemic autoimmune diseases and miRNA-499 gene polymorphism

**Fig 6:**
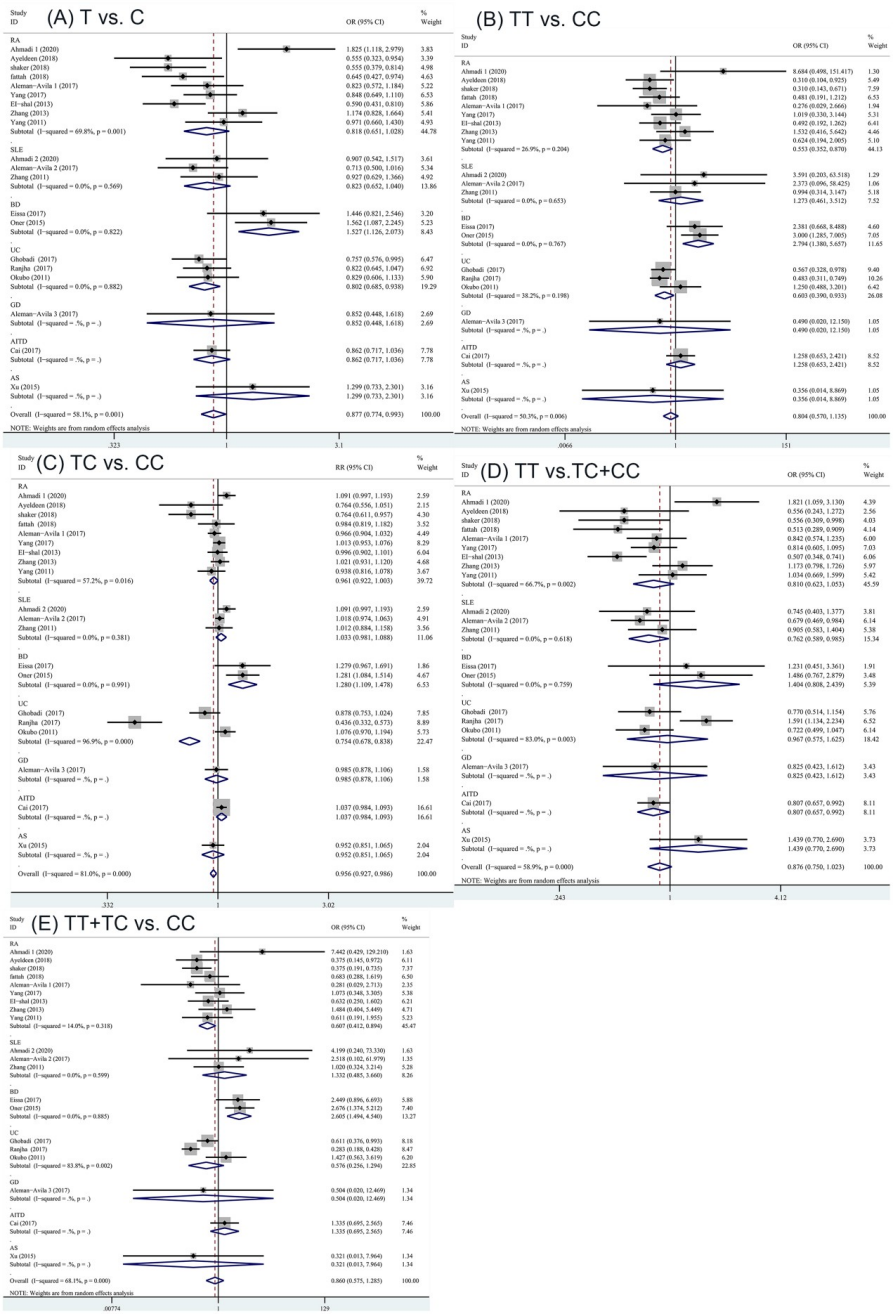
Stratified analysis of the association of the miRNA-499 rs3746444 polymorphisms with the autoimmune diseases by type of autoimmune diseases. (A) T vs. C, (B) TT vs. CC, (C) TC vs. CC, (D) TT vs. TC+CC, (E) TT+TC vs. CC; (RA, rheumatoid arthritis; BD, Behcet’s disease; UC, ulcerative colitis; SLE, systemic lupus erythematosus; GD, Graves’ disease; AITD, autoimmune thyroid diseases; AS, ankylosing spondylitis).

9, 3, 3, and 2 of the included studies analyzed miRNA-499 gene polymorphism in patients with RA, SLE, UC, and BD, respectively. Based on the type of autoimmune diseases, subgroup analysis was performed to assess the relationship between miRNA-499 gene polymorphism and RA, SLE, UC, and BD. In RA studies, there was a significant association in some genetic models (TT vs. CC: OR = 0.553; 95% CI: 0.352, 0.870; P = 0.010; TT+TC vs. CC: OR = 0.607; 95% CI: 0.412, 0.894; P = 0.012). In SLE studies, the result show that there has been a marked association in dominant model(TT vs. TC+CC: OR = 0.762; 95% CI: 0.598, 0.985; P = 0.038). In UC studies, two contrast genetic models (T vs. C and TT vs. CC) showed statistically significant association with random effect model (T vs. C: OR = 0.802; 95% CI: 0.685, 0.938; P = 0.006 TT vs. CC: OR = 0.603; 95% CI: 0.390, 0.933; P = 0.023). In BD studies, four contrast genetic models (T vs. C, TC vs. CC, TT vs. CC and TT+TC vs. CC) showed statistically significant association with random effect model(T vs. C: OR = 1.527; 95% CI: 1.126, 2.073; P = 0.007 TC vs. CC: OR = 2.551; 95% CI: 1.440, 4.520; P = 0.001 TT vs. CC: OR = 2.794; 95% CI: 1.380, 5.657; P = 0.004 TT+TC vs. CC OR = 2.605; 95% CI: 1.494, 4.540; P = 0.001)(Table 4 and Fig 6).

**Table 4:** The result of subgroup analysis by diseases.

rs3746444	No	Genetic model	Association test		Heterogeneity		Prior Probability	
			OR(95% CI)	*p*	I^2^(%)	*p*_H_	0.25	0.1
RA	9	T vs. C	0.818(0.651–1.028)	0.084	69.8	0.001	0.210	0.443
	9	TT vs. CC	**0.553(0.352–0.870)**	**0.010**	28.9	0.204	0.130	0.309
	9	TC vs. CC	0.700(0.486–1.008)	0.055	0.0	0.435	0.215	0.452
	9	TT+TC vs. CC	**0.607(0.412–0.894)**	**0.012**	14	0.318	0.098	0.246
	9	TT vs. TC+CC	0.810(0.623–1.053)	0.115	66.7	0.002	0.272	0.528
SLE	3	T vs. C	0.823(0.652–1.040)	0.103	0.0	0.569	0.243	0.490
	3	TT vs. CC	1.273(0.481–3.512)	0.641	0.0	0.653	0.755	0.902
	3	TC vs. CC	1.547(0.542–4.413)	0.414	0.0	0.516	0.723	0.887
	3	TT+TC vs. CC	1.332(0.485–3.660)	0.579	0.0	0.599	0.746	0.898
	3	TT vs. TC+CC	**0.762(0.589–0.985)**	**0.038**	0.0	0.618	0.119	0.288
UC	3	T vs. C	**0.802(0.685–0.938)**	**0.006**	0.0	0.882	0.017	0.050
	3	TT vs. CC	**0.603(0.390–0.933)**	**0.023**	38.2	0.198	0.175	0.390
	3	TC vs. CC	0.518(0.125–2.147)	0.365	93.6	<0.001	0.750	0.900
	3	TT+TC vs. CC	0.576(0.256–1.294)	0.181	83.8	0.002	0.601	0.819
	3	TT vs. TC+CC	0.967(0.575–1.625)	0.900	83	0.003	0.746	0.898
BD	2	T vs. C	**1.527(1.126–2.073)**	**0.007**	0.0	0.822	0.042	0.116
	2	TT vs. CC	**2.794(1.380–5.657)**	**0.004**	0.0	0.787	0.235	0.480
	2	TC vs. CC	**2.551(1.440–4.520)**	**0.001**	0.0	0.994	0.104	0.258
	2	TT+TC vs. CC	**2.605(1.494–4.540)**	**0.001**	0.0	0.885	0.078	0.203
	2	TT vs. TC+CC	1.404(0.808–2.439)	0.229	0.0	0.759	0.536	0.776
GD	1	T vs. C	0.852(0.448–1.618)	0.624	N/A	N/A	N/A	N/A
	1	TT vs. CC	0.490(0.020–12.15)	0.663	N/A	N/A	N/A	N/A
	1	TC vs. CC	0.620(0.024–16.114)	0.774	N/A	N/A	N/A	N/A
	1	TT+TC vs. CC	0.504(0.020–12.469)	0.675	N/A	N/A	N/A	N/A
	1	TT vs. TC+CC	0.825(0.423–1.612)	0.574	N/A	N/A	N/A	N/A
AITD	1	T vs. C	0.862(0.717–1.036)	0.114	N/A	N/A	N/A	N/A
	1	TT vs. CC	1.258(0.653–2.421)	0.492	N/A	N/A	N/A	N/A
	1	TC vs. CC	1.615(0.824–3.165)	0.162	N/A	N/A	N/A	N/A
	1	TT+TC vs. CC	1.335(0.695–2.565)	0.385	N/A	N/A	N/A	N/A
	1	TT vs. TC+CC	0.807(0.657–0.992)	0.042	N/A	N/A	N/A	N/A
AS	1	T vs. C	1.299(0.733–2.301)	0.039	N/A	N/A	N/A	N/A
	1	TT vs. CC	0.356(0.014–8.869)	0.529	N/A	N/A	N/A	N/A
	1	TC vs. CC	0.238(0.009–6.116)	0.386	N/A	N/A	N/A	N/A
	1	TT+TC vs. CC	0.321(0.013–7.964)	0.488	N/A	N/A	N/A	N/A
	1	TT vs. TC+CC	1.439(0.770–2.690)	0.254	N/A	N/A	N/A	N/A

### 3.4 FPRP Analyses

To further minimize random errors to confirm the positive association between miRNA-499 rs3746444 and a variety of autoimmune diseases, we performed an FPRP analysis. The results are shown in Tables 2 and 4. With the assumption of a prior probability of 0.25, the most FPRP values of Positive association results were <0.2, implying that these significant associations were notable. However, in the subgroup analysis, the FPRP values of BD under the TT vs. CC model(FPRP = 0.235) were >0.2, showing the associations were not noteworthy.

## 4. Discussion

Autoimmune diseases include a wide range of human diseases, which are characterized by loss of immune tolerance to autoantigens, and the presence of autoreactive immune cells and/or autoantibodies against healthy cells and normal tissues [38]. The etiology and pathogenesis of autoimmune diseases are highly complex, involving genetic susceptibility, environmental factors, and epigenetic changes, and they are still largely unknown [39]. miRNAs are important molecules for maintaining the normal function of the immune system. Research in recent decades has revealed the effects of dysregulated miRNAs in the pathogenesis of autoimmune diseases since they participate in the post-transcriptional regulation of a variety of cellular processes [40, 41]. Some studies found miRNAs can contribute to the initiation of autoimmune diseases by regulating autophagy [42, 43].

The miRNA-499 gene is located on chromosome 20, which regulates the expression of its target genes including IL-2, IL-6, IL-17RB, IL-21, IL-23a, and so on [44–46]. IL-17RB, IL-23a, IL-21, and IL-6 are significantly related to autoimmune diseases [47–49]. Regulatory factor X 4 is also a target of miRNA-499, and it can affect the expression of human leukocyte antigen-DRB1(HLA-DRB1). While related research indicated HLA-DRB1 is closely related to autoimmune diseases [16]. In addition, miRNA-499 can affect the production of anti-cyclic citrullinated peptidem antibody by regulating the expression of the peptidyl argininedeiminase type 4 gene [50, 51]. And miRNA-499 gene plays role in several inflammatory diseases through TLR and NF-KB signaling [52]. Therefore, miRNA-499 rs3746444 polymorphism may affect an individual’s susceptibility to autoimmune diseases because SNPs often affect the function of miRNAs.

Numerous case-control studies suggest that the miRNA-499 rs3746444 polymorphism is related to a variety of autoimmune diseases [12–14], and the previous meta-analysis showed that this polymorphism is related to the increased risk of RA [14, 17, 18]. The meta-analysis by Xiao et al. included ten studies published that just focused on RA before 2019. The results suggested a significant association between miRNA-499 rs3746444 polymorphisms and RA risk [17]. Each autoimmune disease has its characteristics, but recent researches found that some autoimmune diseases share several common mechanisms [53–55], and genetic studies have shown that different autoimmune diseases can largely share the same genetic background [56–58]. People with an autoimmune disease are at risk for another autoimmune disease [58], and their family members are at increased risk for more than just this autoimmune disease [59, 60]. Some shared features and familial aggregation imply the necessary link among autoimmune diseases. We imagined whether the correlation between miRNA-499 rs3746444polymorphism and certain autoimmune diseases can be extended to multiple autoimmune diseases represented by RA. In addition, some related case-control studies which were not included in the previous meta-analysis were published in recent years. Based on this assumption and the shortcomings of the previous meta-analyses, We conducted this meta-analysis.

Ultimately, Seventeen articles (twenty studies) with 4,376 cases and 4,991 controls were included in our analysis. Our results suggested that the C allele of miRNA-499 rs3746444 T/C variant was associated with an elevated risk of autoimmune diseases under the allelic model. In addition, by subgroup analysis, we found that T allele in the Asian population and TT genotype in the Caucasian population were protective factors for predisposition to autoimmune diseases, respectively under allelic model (T vs. C) and dominant model (TT vs. TC+CC). Subgroup analysis by disease types showed the T allele and TT genotype behave as protective factors for predisposition to RA, SLE, and UC populations under some genetic models. Conversely, both case-control studies on BD showed that the T allele of miRNA-499 rs3746444 T/C was linked to an increased risk of BD.

There are some previous genome-wide association studies (GWAS) relevant to Behcet’s Disease [61], rheumatoid arthritis [62], and systemic lupus erythematosus [38]. The results of the study did not find a significant relationship between miRNA-499 gene polymorphism and these diseases, which is not consistent with the findings of our meta-analysis. One of the reasons may be that most GWAS pay more attention to European populations. This meta-analysis contains a large number of Asian populations and focuses on many types of autoimmune diseases. Another reason is the limitations of GWAS itself. The GWAS explains only a modest fraction of the missing heritability and GWAS cannot identify all genetic determinants of complex traits [63]. The next step may require more GWAS and case-control studies to explain the difference between the results.

There are several limitations in our meta-analysis. First, there were only three studies on ulcerative colitis (UC) and two studies on Behcet’s disease (BD). Only one study each was related to autoimmune thyroid diseases, ankylosing spondylitis (AS), and Graves’ disease (GD). Because of the limited number of studies and types of autoimmune diseases, the results we have obtained so far still need to be updated in the future. Second, there were different geographic areas and genetic backgrounds in the included studies. The environmental factors might influence the pooled results. Moreover, we could not analyze the gene-environment interactions to investigate the association between rs3746444 polymorphism and autoimmune disease risk.

Despite the limitations, there are some strengths in our meta-analysis. Seventeen articles (twenty studies) were included in our article. The number of studies was significantly greater than that in other meta-analyses published before. This could increase the statistical power in the overall analysis. Seven studies were related to RA in our analysis. There was sufficient data to fully confirm the association of RA and rs3746444 polymorphism.

In conclusion, this meta-analysis suggested that the miRNA-499 rs3746444 polymorphism was associated with an elevated risk of autoimmune diseases in the overall analysis as well as Caucasian and Asian populations. Moreover, significant associations were also found in stratified analysis in Behcet’s disease, rheumatoid arthritis, systemic lupus erythematosus, and ulcerative colitis populations.

## Supporting information

S1 File Data for analysis. (XLS)

S2 File PRISMA checklist. (DOCX)

S1 Checklist Meta-analysis on genetic association studies checklist. (DOCX)
